# The Back Bone

**Published:** 1873-09

**Authors:** 


### THE BACK BONE

is called the "spinal column," and consists of twenty-six pieces, seven for the neck, or "cerebral" portion, the "dorsal," or back proper, includes twelve; the "lumbar" region, or loins, takes five parts; below these are two large bones, called "sacrum" and "coccyx." This latter, or the lowest bone of all, is the base or resting point of the spinal column. In sitting down, with the space of several inches between the back of the seat and the person, the extremity of the coccyx, being covered with almost nothing but skin, frequently gets sore from its contact with the hard seat, and sometimes causes considerable discomfort every time a person sits down; hence children especially should be taught that the proper method in sitting down is to have the lower part of the back touching the back of the seat, then the point of the coccyx does not touch the seat at all, being protected by the natural pads or cushions of the body. Sometimes persons apply to a physician for relief when the extremity of the coccyx is so tender and inflamed that the slightest touch causes exquisite pain. Another advantage of sitting as suggested is that it cultivates an erect position of body, which so much adds to the "presence" of man or woman.

If the spinal column was made of but one bone we could not bend in any direction, nor turn the head; besides, every step we would take would so jar the brain as to make walking intolerable.

Between each of the twenty-four pieces of back bone proper is a soft cushion, made of cartilage, which has a spring in it like India rubber. This breaks the jar or jolt every time the heel strikes the ground, and prevents its being communicated to the brain. When a person sits and walks in an erect position always, these cushions are pressed upon equally on all sides; but if a person bends to one side habitually, as those do who sit with one shoulder lower than the other, the cushion is more pressed on that side, and grows thinner, not only by the pressure but by the absorption of its particles, which always follows pressure.

On the other hand, the opposite side, being less pressed, is thicker—and, in addition, new matter is deposited faster and increases the thickness—thus aiding to tilt the body over in the opposite direction, making it more and more crooked.

If a person bends forward a great deal this gives the stoop shoulder, and the head is more and more thrown forward, and as old age comes on they cannot bend back—cannot straighten themselves—because the particles which in younger years were compressible gradually harden into a kind of lime or bone.

But if persons will habitually sit erect in youth and adult life the cushions begin to get bony, and it is just as difficult for them to sit crooked as for others to sit straight. If persons become "stoop shouldered" the chest is not developed, the lungs do not have an opportunity of expanding healthfully, with the result that consumptive disease is invited and life is shortened many years. It greatly aids in getting an erect position in sitting by adopting the suggestions already made. When sitting down, let the lower part of the back of the body press against the back of the seat, and, in addition, compel the shoulders to touch the back of the seat. The easiest plan to walk erectly is to fix the eye upon something ahead which is higher than itself, or the top of the hat or bonnet of a person ahead of you, or on the eave of a house or top of a tree. This throws the chin above a horizontal line slightly, and the person walks easily, without any effort or strain whatever, the toes are thrown out, and the chest is protruded instinctively, giving an easy, unconstrained and graceful carriage, which is well worth striving for.

